# Awareness Among Dental Practitioners in India Regarding the Caries Assessment Tools Used in Epidemiological Survey: A Cross-Sectional Study

**DOI:** 10.7759/cureus.84805

**Published:** 2025-05-25

**Authors:** Sweta Rastogi, Shahnaz Nabi, Neelam Singh, Sanjay Miglani, Mohd I Ansari

**Affiliations:** 1 Conservative Dentistry and Endodontics, Faculty of Dentistry, Jamia Millia Islamia, New Delhi, IND

**Keywords:** caries assessment spectrum & treatment index, caries assessment tools, caries prevalence, decayed missing and filled teeth (dmft) index, dental caries, epidemiological survey, icdas-ii, pufa index

## Abstract

Introduction

Dental caries is one of the major health problems that researchers and clinicians are trying to tackle at a global level. To halt the progression of disease, it is important to assess dental caries prevalence at both the individual and community levels. Therefore, we have conducted this study to gather information regarding the caries assessment tools, Decayed, Missing, and Filled Teeth (DMFT) index, International Caries Detection and Assessment System (IDCAS II) index, Pulpal Involvement, Ulceration, Fistula, and Abscess (PUFA) index, Caries Assessment Spectrum and Treatment (CAST) index, which are used in epidemiological surveys by dental practitioners. Diagnosis right at the inception of any disease is a first step toward its prevention.

Methodology

This was a questionnaire-based descriptive cross-sectional study. It was carried out in an online mode by circulating a Google Form (Google, Inc., Mountain View, CA) among 244 dentists through various forms of social media availability. Data confidentiality was maintained by the investigators of the study. Data obtained from the study were subjected to statistical analysis using SPSS (IBM SPSS Statistics for Windows, IBM Corp., Version 21, Armonk, NY). The chi-square test of proportion was used for inferential statistics.

Results

One hundred fifty-five (63.5%) respondents believed that a caries assessment tool used in epidemiological surveys should record all the stages of dental carious lesions, right from incipient caries up to the stage of pulpal involvement due to caries. Two hundred seventeen (88.9%) participants believed that DMFT is the most commonly used and the oldest index due to its simplicity and ease of application. Assessing the knowledge regarding the ICDAS II tool, 63 (25.8%) of respondents did not know the carious lesion parameters that could be measured using this index. Only a handful of respondents (40 (16.4%)) were fully aware of the objective of the PUFA index to track the progression of untreated carious lesions. Familiarity with the CAST index was extremely low, as barely 87 (35.7%) participants suggested that it could record the full spectrum of dental illness, and 122 (50%) had no knowledge pertaining to the index. Ninety-one (37.3%) and 84 (34.4%) participants were of the opinion that DMFT and ICDAS II, respectively, were the most suitable tools to record dental caries prevalence in epidemiological surveys. Two hundred twenty-one (90.6%) participants advocated the need for conducting regular training programs to apprise dental practitioners in reference to the caries assessment tools used in epidemiological surveys.

Conclusion

This study enlightened us about the extent of knowledge among dental practitioners toward the different caries assessment tools used in epidemiological surveys. Therefore, this information will act as a starting point for planning seminars and workshops to educate dentists about caries assessment tools at an expansive level. It will also take a further step toward caries prevention by enabling the health policymakers to plan appropriate preventive and curative measures applicable globally.

## Introduction

Dental caries remains a prevalent public health issue, affecting individuals across all age groups globally [1]. According to the World Health Organization (WHO) Global Oral Health Status Report 2022, dental caries is the most widespread non-communicable oral disease, with a prevalence of untreated dental caries in permanent teeth of more than two billion people and in deciduous teeth of around 514 million children worldwide [2].

The effective management and prevention of dental caries depend significantly on accurate diagnosis and assessment, which can be achieved through epidemiological surveys [3].

Diagnosis and assessment of dental carious lesions involve both conventional clinical visual/tactile examination techniques as well as electronic equipment or digital technology. The aforementioned techniques form the foundation of dental caries epidemiological surveys [4].

These surveys are essential for understanding the distribution and determinants of caries, thereby guiding public health interventions and policies [5]. To collect reliable data, various caries assessment tools are employed, including the Decayed, Missing, and Filled Teeth (DMFT) index, International Caries Detection and Assessment System (ICDAS II) index, Pulpal Involvement, Ulceration, Fistula, and Abscess (PUFA) index, and Caries Assessment Spectrum and Treatment (CAST) index [6,7].

The DMFT index, modified by the WHO, is the oldest caries assessment tool being used globally. It evaluates cavitated dentin carious lesions, restored teeth, and missing teeth due to dental caries [8]. The lesions that have progressed to the pulp or those restricted to enamel are not documented via the DMFT index, leading to an underestimation of disease prevalence [8]. Due to a paradigm shift toward preventive care and minimally invasive dentistry, where detection of enamel carious lesions was equally important, the genesis of the other alternative indices was led [9].

ICDAS II is a two-digit classification system developed to overcome the drawbacks associated with the DMFT index. The first digit denotes the tooth surface suggestive of any restorations or sealants, and the second digit provides a detailed overview (three stages each) of enamel as well as dentin caries lesions [8]. The breakthrough offered by the ICDAS II instrument was its capability to detect even the initial changes at any dental surface due to caries, as well as distinguish and monitor the progression of different stages of caries in enamel and dentin [10,11]. This index did not gain enough popularity for use in epidemiological surveys as it is very time-consuming to carry out, complex to analyze, and fails to measure the progression of dental caries into pulp or abscess stage [9,12].

The PUFA index was formulated to deal with the shortcomings of DMFT and ICDAS II so as to facilitate the evaluation of sequelae and severity of untreated carious lesions extending to the pulp and supporting tissues only [13]. However, the PUFA index fulfilled the lacunae of an instrument that could record the burden of advanced stages of untreated carious lesions prevalent especially in middle- and low-income countries. Nevertheless, DMFT, ICDAS II, and PUFA instruments, when used individually, could not satisfactorily assess the real spectrum of the impact of dental caries in a given population, giving rise to the need for a more comprehensive index. Therefore, the CAST index came into existence in 2011 to be used in epidemiological surveys worldwide [14].

The CAST index was obtained by combining a few sections from the WHO and ICDAS II instruments along with the scoring of pulp exposure, abscess, and fistula as proposed by the PUFA index [11,14]. Hence, the CAST index evaluates different stages in a tooth right from health to disease, which is inclusive of the absence of dental caries, preventive measures taken against caries (sealants), the presence of restorations (direct/indirect), carious lesions extending to enamel, dentin, pulp, and supporting tissues of teeth (abscesses/fistulae), and eventually the loss of a tooth [14]. The scoring of the caries spectrum in the CAST index is done in a hierarchical manner, which implies that the higher the score, the more severe the condition. The CAST index is an uncomplicated and easy-to-use caries assessment instrument in any setting, as it does not necessitate repeated drying of the tooth surface prior to evaluation [14].

Despite the availability of these tools, there is considerable variation in their use among dental practitioners throughout the country. This variation can be attributed to differences in training level, awareness, and the perceived practicality of each caries assessment tool [6]. It affects the quality of data collected and influences the effectiveness of caries management and prevention strategies in clinical practice [5].

The objective of this study was to assess the knowledge and awareness levels of dental practitioners regarding various caries assessment tools used in epidemiological surveys. This gap in knowledge can lead to inconsistencies in caries management and prevention strategies, ultimately affecting patient outcomes. The survey questions are designed to explore practitioners' familiarity with these tools, their application, and the perceived need for further education to optimize caries assessment and management [5,15]. By identifying gaps in knowledge and areas for improvement, this study highlights the need for standardized training and education to enhance the use of these tools in research and clinical settings.

## Materials and methods

A descriptive cross-sectional study was conducted to assess knowledge of caries assessment tools used in epidemiological surveys among dental practitioners in India. It was a questionnaire-based study conducted between 15 June and 15 September 2024 in an online format via a Google Form (Google, Inc., Mountain View, CA). The questionnaire was circulated among the dentists working in different parts of India using various social media platforms (Appendices).

The face and content validity of the questionnaire were evaluated by five subject experts. It was observed that the questionnaire had good face and content validity.

For evaluating the reliability of the questionnaire, intra-observer reliability was evaluated in 10 participants at different time intervals. It was observed that the intraclass correlation coefficient (ICC) was 0.92, depicting a strong ICC value.

Ethical aspects

The proposal of the study was approved by the Internal Research Review Committee, Faculty of Dentistry, Jamia Millia Islamia, New Delhi, under the number FOD/IRRC/140/24062024/F.

Eligibility criteria

The study included the general dentists, academicians, and postgraduates who were willing to participate in the study. Informed consent was obtained from the participants, and confidentiality of the responses was maintained.

Sample size estimation 

The sampling methodology employed was non-probability convenience sampling to select the participants. Sample size was estimated using OpenEpi Version 3 (Andrew G. Dean, Kevin M. Sullivan, and Minn Minn Soe, Emory University, Rollins School of Public Health, Atlanta, GA), open-source calculator expecting 80% prevalence of awareness among dentists regarding caries assessment tools as per previous literature, an absolute precision of 5% and a 95% confidence interval, a sample size of 244 is found to be sufficient [16].

The formula used for sample size estimation was: n = [DEFF*Np(1-p)]/[(d2/Z21-α/2*(N-1)+p*(1-p)], with p: expected prevalence = 0.80 or 80%; d: absolute precision required on either side of prevalence = 0.05 or 5%; and Z1-α = 1.96 (DEFF: design effect).

Study proforma and data collection

The survey was circulated among dentists via diverse forms of social media platforms. The questionnaire was prepared primarily in the form of a Likert scale and had 24 questions, divided into three sections. The first section obtained the consent of the participants; the second section collected the demographic details of the participants, while the third section had 20 questions about the knowledge and applicability of caries assessment tools.

Statistical analysis

The data were obtained and entered into Microsoft Excel Version 13 (Microsoft Corp., Redmond, WA). The data were analyzed using SPSS (IBM SPSS Statistics for Windows, IBM Corp., Version 21, Armonk, NY). For the categorical variable, frequency and percentage of the data were obtained. The chi-square test of proportion was applied to evaluate the difference in the proportion. All the statistical tests were applied keeping the confidence interval at 95%, and (p < 0.05) is considered to be statistically significant.

## Results

The demographic data of the participants showed that a majority (71 (29.1%)) were academicians working in government or private institutions, followed by 61 (25.0%) private practitioners and 55 (22.5%) postgraduate students (p < 0.001). The specialty-wise distribution comprised 104 (42.6%) participants from Conservative Dentistry and Endodontics, 14 (5.7%) from Orthodontics and Dentofacial Orthopedics, and 18 (7.4%) from Pedodontics and Preventive Dentistry; the significant p-value is less than 0.001. Regarding the years of practice, 104 (42.6%) of the total 244 respondents have been in practice for more than 10 years, while 77 (31.6%) had less than five years of experience (p < 0.001), as depicted in Table 1.

**Table 1:** Q2-Q4 demonstrate the demographic data pertaining to the participants of this study

		Frequency	Percent	Chi square (ꭓ^2^)	p-value
Q2. What is your current area of practice?	Postgraduate student	55	22.5	31.082	<0.001
	Academician in government/private institution	71	29.1		
	Private practitioner	61	25.0		
	Both b and c	31	12.7		
	Others	26	10.7		
Q3. Please specify your specialty, if any:	Conservative Dentistry and Endodontics	104	42.6	292.164	<0.001
	Orthodontics and Dentofacial Orthopedics	14	5.7		
	Pedodontics and Preventive Dentistry	18	7.4		
	Prosthodontics and Crown and Bridge	12	4.9		
	Oral and Maxillofacial Surgery	15	6.1		
	Oral Medicine and Radiology	5	2.0		
	Periodontics	9	3.7		
	Public Health Dentistry	10	4.1		
	Oral Pathology and Microbiology	8	3.3		
	General practitioner	49	20.1		
Q4. For how many years have you been practicing dentistry?	<5 years	77	31.6	10.680	<0.001
	5-10 years	63	25.8		
	>10 years	104	42.6		
	Total	244	100.0		

Regarding dental caries and assessment of caries, most, 144 (59.0%) respondents, agreed that the polarization of dental caries prevalence is gaining speed in most parts of the world due to increased socioeconomic disparities, and 39 (16.0%) respondents strongly agreed (p < 0.001). Besides, 131 (53.7%) participants believed that epidemiological surveys are of critical importance for maintaining successful caries prevention, while 80 (32.8%) strongly agreed with the statement (p < 0.001). The majority of the participants, 155 (63.5%), believed that a caries assessment tool should record all stages of carious lesions, from incipient caries to pulp involvement (p < 0.001), as depicted in Table 2. 

**Table 2:** Distribution of responses obtained from study participants for Q1-Q5 from section 3

		Frequency	Percent	Chi square (ꭓ^2^)	p-value
Q1. Do you agree that dental caries prevalence is becoming increasingly polarized due to socioeconomic disparity worldwide?	Strongly disagree	24	9.8	238.746	<0.001
	Disagree	14	5.7		
	Neither agree nor disagree	23	9.4		
	Agree	144	59.0		
	Strongly agree	39	16.0		
Q2. Do you believe that epidemiological surveys are need of the hour for effective caries prevention at community level?	Strongly disagree	18	7.4	248.582	<0.001
	Disagree	3	1.2		
	Neither agree nor disagree	12	4.9		
	Agree	131	53.7		
	Strongly agree	80	32.8		
Q3. Objective of a caries assessment tool is to record:	Incipient caries	55	22.5	217.311	<0.001
	Cavitated carious lesions	33	13.5		
	Pulp involvement	1	0.4		
	All of the above	155	63.5		
Q4. Which are the caries assessment tools that you are aware of? (Can choose more than one option)	Decayed, Missing, and Filled Teeth (DMFT) index	67	27.5	251.344	<0.001
	International Caries Detection and Assessment System (IDCAS II) index	1	0.4		
	Pulpal Involvement, Ulceration, Fistula, and Abscess (PUFA) index	0	0		
	Caries Assessment Spectrum and Treatment (CAST) index	1	0.4		
	None of the above	2	0.8		
	All of the above	68	27.9		
	Multiple options selected	105	43.0		
Q5. Has the DMFT index been used for the past 75 years because of its simplicity and ease of application?	Yes	217	88.9	340.484	<0.001
	No	7	2.9		
	Do not know	20	8.2		

About the awareness of caries assessment tools, 68 (27.9 %) of the respondents selected all, indicating familiarity with DMFT, ICDAS II, and CAST indices (p < 0.001). Also, most of the responding individuals in the survey reported that the DMFT index is easy and simple to apply. In this regard, 217 (88.9%) did recognize this, while 118 (48.4%) thought it required an alternative index due to its inability to distinguish between severities of dentin carious lesions, being a prime concern highlighted by respondents. Regarding their knowledge of the ICDAS II, 101 (41.4%) reported partial knowledge, but 80 (32.8%) indicated full awareness. The p-value in each of these cases was less than 0.001, as depicted in Tables 2-3.

**Table 3:** Distribution of responses obtained from study participants for Q6-Q10 from section 3

		Frequency	Percent	Chi square (ꭓ^2^)	p-value
Q6. Can you identify some reasons that gave rise to the need for more comprehensive indices as an alternative to DMFT? (Can choose more than one option)	Does not record a pre-cavitated lesion/enamel carious lesion/incipient caries	32	13.1	176.861	<0.001
	Does not record dentin carious lesion	6	2.5		
	Leads to overestimation of prevalence and severity of caries	15	6.1		
	Does not distinguish between severity of carious lesions	73	29.9		
	Multiple options selected.	118	48.4		
Q7. Are you familiar with the ICDAS tool developed to address shortcomings of DMFT?	Fully aware	80	32.8	8.910	<0.001
	Partially aware	101	41.4		
	Unaware	63	25.8		
Q8. Which parameters regarding dental carious lesions can be measured using ICDAS?	Surface changes	39	16.0	184.525	<0.001
	Histological depth	2	.8		
	Severity	17	7.0		
	All of the above	123	50.4		
	Do not know	63	25.8		
Q9. According to you, what is the advantage favoring use of ICDAS tool?	Detects incipient occlusal caries with low specificity and sensitivity	26	10.7	222.180	<0.001
	Differentiates between cavitated and non-cavitated lesions	28	11.5		
	Provides comprehensive data only on dentin carious lesion severity	8	3.3		
	All of the above	116	47.5		
	None of the above	3	1.2		
	Do not know	63	25.8		
Q10. Select the correct statements pertaining to ICDAS II tool requiring need for an additional index: (Can choose more than one option)	A cumbersome and time-consuming tool as it necessitates drying of the tooth surface	17	7.0	198.836	<0.001
	Complex three digit system and difficult to analyze	9	3.7		
	Does not document the progression of carious lesion to pulp or abscess stage	20	8.2		
	All of the above	66	27.0		
	None of the above	9	3.7		
	Do not know	98	40.2		
	Multiple options selected	25	10.2		

Only 28 (11.5%) respondents were of the opinion that ICDAS II had a remarkable advantage in differentiating between cavitated versus non-cavitated carious lesions. Further, 63 (25.8%) responses were undecided. The percentage of the true answers was statistically significant (p < 0.001). Ninety-eight (40.2%) respondents had no idea pertaining to the shortcomings of ICDAS II, giving rise to the need for an additional index (p < 0.001). Similarly, 112 (45.9%) participants had partial knowledge of the PUFA index that charts the progress of carious lesions that are left untreated (p < 0.001), and 99 (40.6%) found that it was feasible to use the PUFA index in order to appraise the prevalence and severity of oral conditions as a result of caries that have been left untreated (p < 0.001), as depicted in Tables 3-4.

**Table 4:** Depicts distribution of responses obtained from study participants for Q11-Q15 from section 3 CAST, Caries Assessment Spectrum and Treatment; DMFT, Decayed, Missing, and Filled Teeth; IDCAS II, International Caries Detection and Assessment System; PUFA, Pulpal Involvement, Ulceration, Fistula, and Abscess

		Frequency	Percent	Chi square (ꭓ^2^)	p-value
Q11. Are you aware of the PUFA index developed to track the progression of untreated carious lesion?	Fully aware	40	16.4	33.967	<0.001
	Partially aware	112	45.9		
	Unaware	92	37.7		
Q12. Is it feasible to use PUFA index to assess the prevalence and severity of oral conditions resulting from untreated caries?	Very feasible	24	9.8	177.844	<0.001
	Feasible	99	40.6		
	Fairly feasible	80	32.8		
	Less feasible	16	6.6		
	Not feasible	25	10.2		
Q13. Is it necessary to use PUFA index in conjunction with ICDAS or DMFT for a comprehensive assessment of dental caries?	Yes	133	54.5	85.680	<0.001
	No	17	7.0		
	Do not know	94	38.5		
Q14. Is CAST index an effective alternative to ICDAS II, PUFA, and DMFT indices individually?	Strongly disagree	5	2.0	284.852	<0.001
	Disagree	11	4.5		
	Neither agree nor disagree	139	57.0		
	Agree	79	32.4		
	Strongly agree	10	4.1		
Q15. Can the CAST index be used to record full spectrum of dental illness?	Yes	87	35.7	47.123	<0.001
	No	35	14.3		
	Do not know	122	50.0		

Conversely, when asked if the CAST index can be an effective alternative to ICDAS II, PUFA, and DMFT indices, only 79 (32.4%) participants agreed, while 139 (57.0%) were neutral (p < 0.001). Moreover, 87 (35.7%) of the respondents believed that the CAST index is able to record the full spectrum of dental illness, but half of the participants were uncertain (p < 0.001). As far as the tools for caries detection are concerned, a meager of 45 (18.4%) respondents agreed to both the statements that ICDAS II and CAST indices detect caries at enamel level, as opposed to the DMFT index, which ultimately leads to an underestimation of caries prevalence compared to the ICDAS II and CAST indices (p < 0.001), as depicted in Tables 4-5.

**Table 5:** Distribution of responses obtained from study participants for Q16-Q20 from section 3 CAST, Caries Assessment Spectrum and Treatment; DMFT, Decayed, Missing, and Filled Teeth; IDCAS II, International Caries Detection and Assessment System; PUFA, Pulpal Involvement, Ulceration, Fistula, and Abscess

		Frequency	Percent	Chi Square (ꭓ^2^)	p-value
Q16. Statement A: Both CAST and ICDAS tools detect caries at the enamel level, unlike DMFT, which detects caries only at the dentin level. Statement B: The DMFT index leads to an underestimation, while the ICDAS and CAST tools lead to an overestimation of dental caries prevalence.	Statement A is false and B is true.	40	16.4	83.172	<0.001
	Statement A is true, and B is false.	41	16.8		
	Both the statements are true.	45	18.4		
	Both the statements are false.	16	6.6		
	Do not know	102	41.8		
Q17. Do you think that CAST index is based on the strengths of ICDAS II and PUFA indices and provides a link to the widely used DMFT index?	Strongly disagree	4	1.6	229.730	<0.001
	Disagree	15	6.1		
	Neither agree nor disagree	120	49.2		
	Agree	91	37.3		
	Strongly agree	14	5.7		
Q18. According to you, which caries assessment tool provides an effective guidance for operative and preventive management of dental caries? (Can choose more than one option)	DMFT	18	7.4	136.352	<0.001
	ICDAS	43	17.6		
	PUFA	6	2.5		
	CAST	16	6.6		
	None of the above	16	6.6		
	All of the above	69	28.3		
	Multiple options selected	76	31.1		
Q19. According to you, which is the most suitable tool for detecting caries prevalence in epidemiological survey?	DMFT	91	37.3	113.418	<0.001
	ICDAS	84	34.4		
	PUFA	10	4.1		
	CAST	41	16.8		
	None of the above	18	7.4		
Q20. Is it necessary to implement regular training programs to educate dental professionals regarding caries assessment tools used in epidemiological surveys?	Yes	221	90.6	360.254	<0.001
	No	7	2.9		
	Do not know	16	6.6		
	Total	244	100.0		

In the last section, 91 (37.3%) respondents agreed that the CAST index is based on the strengths of ICDAS II and PUFA indices, with a link to the DMFT index (p < 0.001). The majority, 76 (31.1%) respondents, preferred multiple tools for an effective caries assessment and management, such as DMFT, ICDAS II, PUFA, and CAST, with a preponderance for ICDAS II. Meanwhile, 91 (37.3%) respondents preferred the DMFT index in detecting the prevalence of caries in epidemiological surveys, closely followed by ICDAS II (84 (34.4%), p < 0.001). Moreover, 221 (90.6%) respondents agreed that regular training programs should be conducted to train dental professionals in caries assessment tools (p < 0.001), as depicted in Table 5.

## Discussion

There is a paradigm shift in the global dental caries pattern wherein developed countries are experiencing a decline in its prevalence, whereas in less developed nations and in emerging economies, it is reaching epidemic proportions. This is polarization of dental caries [17]. “Prevention is better than cure.” This statement holds most true in the context of management of dental caries, wherein, currently, the major emphasis is on prevention rather than operative management of the carious tooth. Early diagnosis forms the cornerstone of preventing any disease. Epidemiological surveys are the need of the hour to assess widespread caries prevalence as well as to guide policymakers to draft adequate treatment and preventive measures to decrease the dental caries burden globally.

Our study results are in accordance with the above statements, as 183 (75%) practitioners agreed with widespread polarization of dental caries, and 211 (86.5%) emphasized the need for epidemiological surveys for global reduction of dental caries burden. A complete caries assessment tool should be able to track the progression of dental caries right from the stage of being incipient to the advanced stages of carious lesion extending to the pulpal and tooth-surrounding tissue. One hundred fifty-five (63.5%) respondents of our survey were also of the opinion that an ideal caries assessment tool should have a broad spectrum. DMFT, ICDAS II, PUFA, and CAST indices are different caries assessment tools available that are used in epidemiological surveys to assess the prevalence of dental caries worldwide.

When asked about their acquaintance with different caries assessment tools, a majority (2/3) were aware of the DMFT index out of the four tools asked. Two hundred seventeen (88.9%) respondents of our study also agreed that the DMFT index by WHO is the oldest index being used globally, as it easily documents the existence or non-existence of dental caries-related tooth loss, restorations, and dentine cavities, and the data it yields can be easily compared [8]. This study also highlights the fact that the DMFT index cannot record the severity of carious lesions that have progressed to the pulp or that are restricted to enamel, leading to an underestimation of disease prevalence [8]. Prevalence for dental caries calculated using the DMFT index includes both the cured and diseased teeth (D-component and M- and F-components, respectively). This method of measuring dental caries is deceptive since it implies that a person can never be free of dental caries, even in the absence of symptoms (i.e., when there are no M- and F-components) [8]. This led to the genesis of alternative indices to record dental caries at the epidemiological level.

A new horizon was offered by ICDAS II, which came into being in 2002 as a result of the International Consensus Workshop on Caries Clinical Trial (ICW-CCT) in Scotland to develop an instrument to record initial stages of carious lesions, which could not be recorded by the DMFT index [10]. It was later modified in 2005 to ICDAS II, a two-digit classification system to facilitate its use in clinical research, clinical practice, dental education, and epidemiological surveys, as the reliability of the earlier assessment instruments was not strong [3,18]. Only 80 (32.8%) participants of the population surveyed were fully aware of this tool, suggesting its lack of popularity among practitioners. This instrument was designed to detect carious lesions at both cavitated and non-cavitated levels in epidemiological surveys with good accuracy and reproducibility [10]. A minor, 28 (11.5%), of the surveyed practitioners appropriately identified this edge offered by ICDAS II.

ICDAS II provided a paradigm shift by measuring surface changes and histologic depth as well as the severity of dentin carious lesions, as agreed by 123 (50.4%) of the respondents in our study. The histologic depth of the lesion and the ICDAS II scores showed a strong and positive correlation. The Ekstrand et al. 1997 classification is the foundation of the histological classification system [11,19]. The shortcomings of the DMFT index may be mitigated by ICDAS II, which differentiates between the three stages of enamel and dentin carious defects. Nevertheless, each tooth must be examined two or three times, and it mandates the use of compressed air to desiccate tooth surfaces. Therefore, using the ICDAS II tool is time-consuming and not cost-effective. ICDAS II also does not record the progression of carious lesions to pulp or abscess stage [20]. Ninety-eight (40.2%) participants were unaware of the shortcomings associated with ICDAS II, which is also indicative of its lack of popularity.

Preliminary focus on prevention and decline of cavitated caries lesions in high-income countries requires an index that distinguishes between the different stages of initial caries lesions, which led to the origin of ICDAS II [21]. However, the scenario in low- and middle-income countries and in deprived sections of high-income countries warrants the need for a diagnostic index that could record advanced stages of untreated carious lesions and their progression to pulp due to the socio-economic deprivation leading to unequal access to health care. Therefore, PUFA came into existence to record very advanced stages of carious lesion progression that were being missed by ICDAS II and the DMFT index [13].

The PUFA index did not gain popularity, which is also reflected in our study, as only 40 (16.4%) participants were fully aware, and 92 (37.7%) were absolutely unaware of this index. Only 24 (9.8%) participants reported extreme feasibility of the PUFA index in recording severe, untreated carious lesions, whereas 25 (10.2%) totally disagreed. One hundred thirty-three (54.5%) respondents in our study believed that the PUFA index is only supplementary to ICDAS II or DMFT for a comprehensive dental caries assessment. PUFA index was initially welcomed, but due to its limited spectrum to measure only a limited aspect of different stages of caries progression, it was considered merely complementary to DMFT or ICDAS II [20].

Shortcomings of PUFA, time-consuming and complex assessment of ICDAS II, and difficulty in comparing the outcomes with DMFT gave rise to the need for a more inclusive index with ease of interpretation [22]. Therefore, a new caries assessment tool called the “CAST index” was developed with a broad spectrum ranging from identifying non-cavitated and cavitated carious lesions to appreciating the consequences of severe, untreated carious lesions, which included pulp involvement, abscesses, and fistulas associated with untreated caries. Also, the index includes the lost as well as restored elements in its results, which makes the outcomes obtained via the CAST index readily comparable to other caries assessment tools like the DMFT index used in epidemiological surveys [14]. Seventy-nine (32.4%) participants of the surveyed population were also of the opinion that the CAST index can be a game changer in the scenario of caries assessment tools being the most comprehensive index of all existing ones. Meanwhile, 122 (50.0%) responders in our study were unaware that the CAST index can record the full spectrum of dental illness and health. 

To do an accurate assessment and implement caries control programs at a massive level, dental practitioners should have an in-depth awareness of the different caries assessment tools used in epidemiological surveys. Only 45 (18.4%) practitioners were of the opinion that both the CAST index and ICDAS II detect caries at the enamel level, leading to increased caries prevalence, unlike DMFT, which detects caries only at the dentin level, leading to an underestimation of caries prevalence. One hundred five (43.0%) respondents in our study approved of the opinion of Frenken et al. [14], who believed that the CAST index can be revolutionary as it is based on the strengths of the ICDAS II, PUFA indices, and DMFT index. The CAST index covers assessment of all stages of carious lesion progression in enamel, dentine, and the pulp, together with teeth sealed, teeth lost due to dental caries, and teeth restored because of dental caries [14].

Management of a carious tooth will be determined upon the stage of the disease and which tooth is affected, and it can range from preventive to operative to removal of the tooth, being the last resort. Early and correct diagnosis will guide the appropriate treatment strategy. Around 69 (28.3%) respondents suggested that all the caries assessment tools would be required to guide both preventive and operative management of carious teeth, whereas 16 (6.6%) believed that none of the tools available could satisfactorily suggest the treatment strategy for carious teeth.

There is polarization of dental caries worldwide primarily due to socio-economic disparities, which is supplemented by the data released by the WHO Global Oral Health Status Report in 2022, which states that dental caries is the most common non-communicable oral disease worldwide [2]. Determining an accurate prevalence of dental caries at a global level via epidemiological survey is of utmost importance in guiding health policymakers in the development of preventive care control programs. Assessment of dental caries prevalence using the DMFT index is ambiguous, as it takes into account not only the diseased teeth but also missing and filled teeth to estimate the caries prevalence. Therefore, it indicates caries experience rather than actual dental caries prevalence in a population [8]. Estimation of dental caries prevalence should be primarily based on dentin carious lesions, and the presence of enamel carious lesions should be noted separately. Calculating enamel carious lesions provides useful information about the extent of the disease and preventive measures to be taken to control it in a given population [23]. 

The CAST index provides an edge over the DMFT index by accepting the repaired and restored teeth as healthy, unlike DMFT, which proves advantageous, especially in epidemiological surveys, to estimate an accurate caries prevalence in a given population [9,14]. Ninety-one (37.3%) and 84 (34.4%) dental practitioners were of the opinion that DMFT and ICDAS II, respectively, are the best tools to record dental caries prevalence in an epidemiological survey, whereas only 41 (16.8%) opted for the CAST index. This could be due to a lack of familiarity with the other tools (PUFA and CAST index), as also depicted by our study. Two hundred twenty-one (90.6%) study participants agreed on the importance of conducting regular training programs to educate dentists regarding caries assessment tools used in epidemiological surveys.

The CAST index presents a promising tool that bridges the limitations of the DMFT, PUFA, and ICDAS indices. Its hierarchical and comprehensive structure allows for the recording of caries across the entire spectrum, from health to severe pathology. Despite its advantages, its limited adoption may be attributed to inadequate awareness and training. The CAST index should not be viewed merely as a replacement, but as a complementary tool, especially useful in epidemiological contexts where both disease prevalence and treatment status must be captured.

Limitations

A major limitation of this study is the use of convenience sampling via social media dissemination, which may have led to a selection bias by overrepresenting dentists who are more digitally active. The study is also subject to self-reporting bias, as participants may have overestimated their familiarity with caries assessment tools. Additionally, there is currently no universally accepted gold standard to objectively measure awareness levels regarding caries indices, which limits the comparability and external validation of the findings. As the survey was distributed via multiple uncontrolled social media platforms, we were unable to determine the exact number of recipients, and thus, the response rate could not be calculated. This limitation restricts the assessment of potential non-response bias.

Future directions

Regular training programs should be conducted for dental practitioners to familiarize them with the ICDAS and CAST indices to focus on the practical application of these indices in clinical settings, emphasizing their advantages over traditional methods and developing concrete interventions, such as curriculum reforms or continuing education, to enhance the knowledge of dental practitioners.

## Conclusions

This survey highlights the skewed familiarity of dental practitioners pertaining to a few caries assessment tools used in epidemiological surveys. Henceforth, it accentuates the importance of conducting frequent training programs at a massive level to spread awareness regarding the different caries assessment tools used in epidemiological surveys. The recent caries assessment tools are not only suggestive of a more accurate caries prevalence, but they also help us to detect caries even at the enamel level. This will pave a pathway for preventive measures to be implemented at a broader level and intern reduce the widespread global burden of dental caries. Our emphasis should always be based on the philosophy that prevention is better than cure. As underlined by the WHO Global Oral Health Status Report 2022, dental caries is the most common oral non-communicable disease globally. Therefore, accurate estimation of dental caries prevalence globally is needed at the hour to guide the health policymakers in implementing and monitoring caries control programs in a widespread manner.

## Appendices

Section 1: Informed consent

This is a questionnaire-based study that is being done purely for academic purposes. Participation in the survey is totally voluntary, and it will take approximately 10 minutes only. The responses obtained will remain confidential with the principal and co-investigators of the study.

Do you wish to participate in this study?

● Yes ● No

Section 2: Demographic details

Q1. Email address:

Q2. What is your current area of practice?

a. Postgraduate student b. Academician in government/private institution c. Private practitioner d. Both b and c e. Others

Q3. Please specify your specialty, if any:

a. Conservative Dentistry and Endodontics b. Orthodontics and Dentofacial Orthopedics c. Pedodontics and Preventive Dentistry d. Prosthodontics and Crown and Bridge e. Oral and Maxillofacial surgery f. Oral Medicine and Radiology g. Periodontics h. Public Health Dentistry i. Oral Pathology and Microbiology j. General practitioner

Q4. For how many years have you been practicing dentistry?

a. <5 years b. 5-10 years c. >10 years

Section 3: Awareness among dental practitioners regarding caries assessment tools

Q1. Do you agree that dental caries prevalence is becoming increasingly polarized due to socioeconomic disparity worldwide?

a. Strongly disagree b. Disagree c. Neither agree nor disagree d. Agree e. Strongly agree

Q2. Do you believe that epidemiological surveys are need of the hour for effective caries prevention at community level?

a. Strongly disagree b. Disagree c. Neither agree nor disagree d. Agree e. Strongly agree

Q3. Objective of a caries assessment tool is to record:

a. Incipient caries b. Cavitated carious lesions c. Pulp involvement d. Abscess/fistula e. All of the above

Q4. Which are the caries assessment tools that you are aware of? (Can choose more than one option) a. DMFT index b. ICDAS c. PUFA index d. CAST index e. None of the above f. All of the above

Q5. Is DMFT index being used for past 75 years because of its simplicity and ease of application? a. Yes b. No c. Don’t know

Q6. Can you identify some reasons that gave rise to the need for more comprehensive indices as an alternative to DMFT? (Can choose more than one option) a. Doesn’t record a pre-cavitated lesion/enamel carious lesion/incipient caries b. Doesn’t record dentin carious lesion c. Leads to overestimation of prevalence and severity of caries d. Doesn’t distinguish between the severity of carious lesions

Q7. Are you familiar with the ICDAS tool developed to address shortcomings of DMFT? a. Fully aware b. Partially aware c. Unaware

Q8. Which parameters regarding dental carious lesions can be measured using ICDAS? a. Surface changes b. Histological depth c. Severity d. All of the above e. Don’t know

Q9. According to you, what is the advantage favoring use of ICDAS tool? a. Detects incipient occlusal caries with low specificity and sensitivity b. Differentiates between cavitated and non-cavitated lesions c. Provides comprehensive data only on dentin carious lesion severity d. All of the above e. None of the above f. Don’t know

Q10. Select the correct statements pertaining to ICDAS II tool requiring need for an additional index: (Can choose more than one option) a. A cumbersome and time-consuming tool, as it necessitates drying of the tooth surface b. Complex three-digit system and difficult to analyze c. Doesn’t document the progression of carious lesion to pulp or abscess stage d. All of the above e. None of the above f. Don’t know

Q11. Are you aware of the PUFA index developed to track the progression of untreated carious lesion? a. Fully aware b. Partially aware c. Unaware

Q12. Is it feasible to use PUFA index to assess the prevalence and severity of oral conditions resulting from untreated caries? a. Very feasible b. Feasible c. Fairly feasible d. Less feasible e. Not feasible

Q13. Is it necessary to use PUFA index in conjunction with ICDAS or DMFT for a comprehensive assessment of dental caries? a. Yes b. No c. Don’t know

Q14. Is CAST index an effective alternative to ICDAS, PUFA, and DMFT indices individually? a. Strongly disagree b. Disagree c. Neither agree nor disagree d. Agree e. Strongly agree

Q15. Can the CAST index be used to record full spectrum of dental illness? a. Yes b. No c. Don’t know

Q16. Statement A: Both CAST and ICDAS tools detect caries at enamel level unlike DMFT, which detects caries only at dentin level. Statement B: DMFT index lead to an underestimation, while ICDAS and CAST tools lead to overestimation of dental caries prevalence: a. Statement A is false, and B is true. b. Statement A is true, and B is false. c. Both the statements are true. d. Both the statements are false. e. Don’t know

Q17. Do you think that CAST index is based on the strengths of ICDAS II and PUFA indices and provides a link to the widely used DMFT index? a. Strongly disagree b. Disagree c. Neither agree nor disagree d. Agree e. Strongly agree

Q18. According to you, which caries assessment tool provides effective guidance for operative or preventive management of dental caries? (Can choose more than one option) a. DMFT b. ICDAS c. PUFA d. CAST e. None of the above f. All of the above

Q19. According to you, which is the most suitable tool for detecting caries prevalence in epidemiological survey? a. DMFT b. ICDAS c. PUFA d. CAST e. None of the above

Q20. Is it necessary to implement regular training programs to educate dental professionals regarding caries assessment tools used in epidemiological surveys? a. Yes b. No c. Don’t know
